# Affect-related synesthesias: a prospective view on their existence, expression and underlying mechanisms

**DOI:** 10.3389/fpsyg.2013.00754

**Published:** 2013-10-18

**Authors:** Nele Dael, Guillaume Sierro, Christine Mohr

**Affiliations:** Institute of Psychology, University of LausanneLausanne, Switzerland

**Keywords:** synesthesia, emotion, affect, appraisal, stability, state dependency, crossmodal

## Abstract

The literature on developmental synesthesia has seen numerous sensory combinations, with surprisingly few reports on synesthesias involving affect. On the one hand, emotion, or more broadly affect, might be of minor importance to the synesthetic experience (e.g., Sinke et al., 2012). On the other hand, predictions on how affect could be relevant to the synesthetic experience remain to be formulated, in particular those that are driven by emotion theories. In this theoretical paper, we hypothesize that a priori studies on synesthesia involving affect will observe the following. Firstly, the synesthetic experience is not merely about discrete emotion processing or overall valence (positive, negative) but is determined by or even altered through cognitive appraisal processes. Secondly, the synesthetic experience changes temporarily on a quantitative level according to (i) the affective appraisal of the inducing stimulus or (ii) the current affective state of the individual. These hypotheses are inferred from previous theoretical and empirical accounts on synesthesia (including the few examples involving affect), different emotion theories, crossmodal processing accounts in synesthetes, and non-synesthetes, and the presumed stability of the synesthetic experience. We hope that the current review will succeed in launching a new series of studies on “affective synesthesias.” We particularly hope that such studies will apply the same creativity in experimental paradigms as we have seen and still see when assessing and evaluating “traditional” synesthesias.

## Introduction to the phenomenon or phenomena

The etymology of synesthesia is derived from the Greek *syn* meaning “together” and *aesthesis* meaning “sensation”; which means literally “union of the senses” (Cytowic, 2002). In this regard, synesthesia can be understood as a phenomenon wherein a simultaneous dual perception arises from a single sensory input (the inducer). The additional percept (the concurrent) belongs either to the same or to a different sensory modality. Prevalence studies could show that one of the most frequent forms of synesthesia is grapheme-color synesthesia (Day, 2005; Rich et al., 2005; Simner et al., 2006). In this form, the perception of an achromatic grapheme (e.g., the letter “p”) is reported to also trigger the subjective perception of a color experience with the respective color perception being idiosyncratic to the individual (e.g., the color “orange”). In some cases, this idiosyncratic color experience is accompanied by texture, shape, and movement qualities (Tyler, 2005). Other forms of synesthesia include reports that seeing or hearing words, numbers, or sounds can activate the simultaneous perception of smells, tastes, and shapes (Cytowic, 2002; Ward and Simner, 2003; Tyler, 2005). Yet, there seem to be countless types of possible synesthetic experiences such as the combinations of time and space (Price and Mentzoni, 2008; Smilek et al., 2010), vision and touch (Banissy and Ward, 2007; Banissy et al., 2009), sound and touch (Beauchamp and Ro, 2008), observing pain in others and feeling this pain oneself (Fitzgibbon et al., 2010), and the attribution of animate-like qualities (e.g., personality, gender) to sequential linguistic units (e.g., letters, numerals) (Simner and Holenstein, 2007)

Reports on synesthesia have existed for centuries (Galton, 1880; Flournoy, 1892; Jewanski et al., 2009). Scientific activities have occurred in waves such as the one around the turn of the last century (Alford, 1918; Mahling, 1926) and a more recent one with the renaissance of the study of cognition (Marks, 1975). Since then, researchers accumulated evidence for the genuineness of the idiosyncratic synesthetic experience (Rich and Mattingley, 2002) using well-established cognitive paradigms (e.g., the Stroop paradigm) and neuroimaging methods. Despite small sample sizes, many of these carefully designed studies were accepted for publication in highly prestigious journals (Dixon et al., 2000; Beeli et al., 2005). Today, the prevalence rate of synesthesia is estimated to be comparable in both sexes and to be evident in around 4% of the population (Ward and Simner, 2005; Simner et al., 2006). This relatively high prevalence rate together with the description of and search for (i) individual differences in the synesthetic experience (Dixon et al., 2004; Skelton et al., 2009; Rouw and Scholte, 2010), (ii) cognitive correlates (Azoulai et al., 2005; Yaro and Ward, 2007; Rothen and Meier, 2009; Banissy et al., 2011; Ward, 2013), and (iii) personality and clinical correlates (Bors, 1979; Forsman, 1993; Ro et al., 2007; Logsdail, 2009; Banissy et al., 2013) contributed to numerous published group studies.

The most studied and thus the most representative type of synesthesia is the developmental one (also known as constitutional, idiopathic or strong synesthesia) (Grossenbacher, 1997; Harrison and Baron-Cohen, 1997; Martino and Marks, 2001). Important to this form is that the experience is present from early childhood, automatic, idiosyncratic, and remains supposedly stable over the course of a lifetime. Apart from the developmental type of synesthesia, the existence of and relationship between different types of synesthesia are discussed (e.g., Rogowska, 2011; Sinke et al., 2012; Ward, 2013), in particular those acquired following particular life events or those affecting the central nervous system more directly. For instance, synesthetic experiences have been reported in association with migraine auras (Podoll and Robinson, 2002; Alstadhaug and Benjaminsen, 2010), post-hypnotic suggestion (Terhune et al., 2010), thalamic lesions (Beauchamp and Ro, 2008), and drug ingestion (Siebert, 1994; Sinke et al., 2012). These latter types, however, lack the features known from the genuine, i.e., developmental, type (automaticity, stability, idiosyncrasy) (Sinke et al., 2012).

## Synesthesias involving emotional experiences

In their comprehensive comparison of developmental, acquired and drug-induced synesthesias, Sinke et al. (2012) discuss interesting phenomenological differences. For instance, external sensory input is the common inducer of developmental synesthesias (vision, audition, touch) with body-related sensory input playing no such role. The authors (as well as the current authors) are unaware of a reported case of synesthesia for which proprioceptive or vestibular information was accompanied by a concurrent synesthetic experience. Pain however does seem implicated in synesthetic experiences (Dudycha and Dudycha, 1935; Fitzgibbon et al., 2010). In one case, a person reports that “each type of pain always gives rise to the same photism with its characteristic form, color or brightness” (p. 58) and varies with the extent of pain (Dudycha and Dudycha, 1935). The same synesthete reports that for dull and throbbing pains, the photism “varies in size and seems to correspond exactly with the affected part” (p. 59). In another case of synesthesia for pain, the respective person reports to empathize with another person's pain and to experience the observed pain, or even the imagined pain, as if it was the own (Bradshaw, 2001; Giummarra and Bradshaw, 2009; Fitzgibbon et al., 2010). Thus, although proprioceptive and vestibular sensory experiences do not seem related to synesthetic experiences, bodily experiences may be related—in particular those with an affective component (own pain or observation of other's pain). This latter observation is important when now commenting on affectivity (Sinke et al., 2012).

On page 1426, Sinke et al. (2012) write about the affectivity of synesthetic experiences. The overall conclusion was that “in genuine synesthesia, the impact of affectivity is low.” The authors state that in developmental synesthesia the content of the synesthetic experience (i) is commonly not affective or emotional in itself, and (ii) is not influenced by current affective or emotional state. Surely, it has been noted anecdotally and experimentally that affective experiences may occur as a secondary effect. For example, an early report (Alford, 1918) describes a synesthete who gave up music because the respective synesthetic experiences were disturbing. Recent studies report that synesthetes react with feelings of “correctness” or “pleasantness” to experiences in which the synesthetic and actual perceived features of a stimulus match (e.g., a grapheme is presented in its synesthetically matching color) and react with feelings of “incorrectness” or “unpleasantness” in the case of mismatches (e.g., a grapheme is presented in a synesthetically non-matching color) (Ramachandran and Hubbard, 2001a; Cytowic, 2002; Callejas et al., 2007). Such subjective experiences yielded psychophysiological correlates (Hochel et al., 2009).

Additional reports would support the possibility that affect is indeed an important sensory experience to developmental synesthesia, and can be so in three possible ways. The affective component is (1) the trigger or inducer (Cutsforth, 1925; Dudycha and Dudycha, 1935; Fitzgibbon et al., 2010), (2) the concurrent (Ramachandran and Brang, 2008; Ramachandran et al., 2012), or (3) neither the inducer nor the direct concurrent, but a moderator or mediator of the synesthetic coupling (Cytowic, 1989; Emrich et al., 2004; Ward, 2004; Simner and Holenstein, 2007; Hochel et al., 2009; Ramachandran et al., 2012). For instance, reactions to words of affective valence as well as to personally known faces triggered emotionally mediated color synesthesia (Ward, 2004). This author replicated previous reports that darker and less saturated colors (e.g., brown, black) tend to be associated with negative emotions and lighter and more saturated colors (e.g., yellow, green, red) tend to be associated with positive emotions (p. 770). In another single case study, independent authors reported that “Pleasing pictures and faces were typically red to R, while repulsive visuals or unpleasant human faces elicited a pale green color in R's mind's eye” (Hochel et al., 2009; p. 705). In other cases, feeling particular textures triggered specific emotions (Ramachandran and Brang, 2008), and emotional situations triggered the synesthetic experience of color (Cutsforth, 1925). Synesthetes are also known for liking or disliking certain letters based on their apparent “personality”—a synesthetic form coined “ordinal linguistic personification” in which letters have a gender, personality, and emotional connotation (Simner and Holenstein, 2007; Smilek et al., 2007). Moreover, overall affective judgment of colors was influenced by the emotional coherence between the stimulus and photism (Hochel et al., 2009). Finally, a synesthete with a diagnosis of Asperger's syndrome better understood his and other persons' emotions when he could associate them to a particular color (Ramachandran et al., 2012).

These reports would suggest that synesthesias involving affect or even concrete emotions are indeed thinkable, and might, as will be argued in the following, imply facets little considered so far. This argumentation will firstly return to the relevance of physical experiences to emotion, before discussing cognitive appraisal in emotion elicitation, the difference between emotion and affect, crossmodal interactions in non-synesthetes, and dynamic changes of sensory experiences with the perceivers' emotional and affective states. Subsequently, we will briefly mention some studies and reports that question the stability and durability of the synesthetic as well as crossmodal experience. Finally, we will conclude that some synesthetic experiences might change in quality or rather in quantity with ongoing emotional and affective states.

## The role of neurophysiological experiences and cognitive appraisal in emotion theories

Emotion researchers frequently disagree on what constitutes an emotion (Scherer, 2005; Mulligan and Scherer, 2012). Historical traditions (James, 1890; Schachter, 1964) and contemporary emotion theories (for example Cacioppo et al., 2000) agree that neurophysiological (e.g., respiratory, cardiac) changes are crucial to emotion processing (for a review, see Kreibig, 2010). Such changes are considered essential to mobilize an organism (e.g., a human) to respond effectively to a given situation. For instance, back in my neighborhood, I am driving around in my car to find a parking space. In front of me, I suddenly see a ball that is rolling onto the street. In this situation, most people are quickly alarmed, experiencing neurophysiological changes that prepare them for action such as to brake hard to avoid the moving object. Our heart rate might increase, our breathing becomes faster and our hands are getting sweaty. The activated peripheral subsystems (cardiovascular, respiratory, electrodermal) respond instantly to situations of potential danger. Yet, the potential danger is not only about the rolling ball, but also about the possibility that a person, perhaps a child, suddenly appears running behind the ball. This possibility, however, goes beyond the perception of the actual situation, it relates to our knowledge of the world and our appraisal of the situation. Indeed, our neurophysiological changes might be particularly strong because hitting the imagined child is much more worrisome than hitting the ball. Thus, the emotional experience is the result of situational elaboration or appraisal, rather than the mere processing of sensory input.

In this regard, it is important to mention that such neurophysiological changes do not sufficiently explain the full emotion process. Firstly, most neurophysiological changes are not specific to a given emotion. Second, the same neurophysiological changes do not necessarily lead to the same subjectively experienced emotion or behavioral response (Cacioppo et al., 2000). While our ball scenario indicated possible danger resulting in a fear or alarm reaction, similar activations of peripheral subsystems (cardiovascular, respiratory, electrodermal) can also accompany the experience of anger (Kreibig, 2010). The latter may occur when one circles around one's neighborhood without finding a free parking space. Needless to say, the fear reaction is subjectively quite unlike the anger reaction despite both being about obstructing events. It has long been understood that the activation of the sympathetic nervous system prepares the organism for both fight and flight responses (Cannon, 1929; Bradley and Lang, 2000). Neurophysiological changes might thus be necessary to have emotional experiences and reactions, but they are largely emotion unspecific. Also, the same emotion can be associated with different patterns of neurophysiological changes (Cacioppo et al., 2000).

Crucial is the way an individual cognitively processes a situation that leads to a behavioral response. When seeing that a ball is rolling onto the street, one individual might speed up to quickly pass the scene of potential danger, while another might brake hard to avoid reaching the scene of potential danger. Also, the lack of a parking space may facilitate an anger response when the driver is in a hurry, and especially so when the driver blames someone else (a badly parked car occupies two parking spaces). Thus, the same event might trigger different emotional experiences and behaviors both between individuals and within individuals at different time points depending on how the individual appraises the event. “Because appraisals intervene between situations and emotions, *different individuals who appraise the same situation in significantly different ways will feel different emotions; and a given individual who appraises the same situation in significantly different ways at different times will feel different emotions*” (Roseman and Smith, 2001; p. 6).

Accordingly, emotions are not “simple” sensory experiences of neurophysiological change, but involve cognitive processes contributing to the elicitation, differentiation, and regulation of emotions (Moors, 2009). Emotions are not directed toward random events or objects. On the contrary, emotional reactions are mainly intentional in that they are directed toward or are about something specific: I am afraid of hitting the child, I am angry at the driver blocking two parking spaces, etc. Emotional intentionality implies again that a given situation or object has gone through some form of prior evaluation, however, minimal. Indeed, in cognitive appraisal accounts (e.g., Scherer, 2001), appraisals range from low level relevance detection (e.g., the rolling ball as a sudden and novel stimulus demanding attention) to higher level goal congruency processing (e.g., lack of parking spaces as an obstacle to relax at home after a demanding working day), coping potential (e.g., evaluating the time needed to stop the car before hitting a potentially appearing child), to the very high level evaluation of the social context with respect to personal and cultural standards (e.g., social evaluation if having hit a playing child) (for reviews, see Roseman and Smith, 2001; Moors, 2009).

Importantly, cognitive appraisal theories would speak against a widely held assumption of modularity in emotion processing such as in discrete emotion theories (Ekman, 1992). Modularity here indicates that an emotion might represent a unique and discrete response pattern that was triggered by a specific affect program, established evolutionarily. This modularity was suggested for prototypical or basic emotions of anger, fear, sadness, disgust, and happiness (Ekman, 1999). For instance, fear would be processed by a discrete neural network in the same way as vision or audition would be processed by discrete visual and auditory neural networks, respectively (Ohman and Mineka, 2001). The above mentioned appraisal rationale would, however, imply that emotional experiences and responses are not simply the result of modular processes that are automatically elicited by the objective properties of a given event or object. Rather, emotional experiences and responses would result from an individual's situational appraisal of a given situation. The quality (type) and quantity (intensity) of the experiences are likely characterized by non-specific neurophysiological changes in conjunction with specific event appraisals.

Acknowledging the relevance of such cognitive appraisal mechanisms, we equally acknowledge the possibility that these mechanisms lead to systematic differences in the synesthetic experience. In other words, the fact that the same stimulus can lead to qualitatively and quantitatively different affective experiences both within and across individuals may contribute to a potential dynamism of the synesthetic experience (see below). Moreover, the inconsistency with which objective properties of specific stimuli and events are linked with subjective affective experiences may have led to an underestimation of the prevalence of emotional synesthesias. The emotion literature would point toward consistent couplings of affective experiences to a synesthete's *appraisal* of the stimulus. For instance, Ward (2004) reported that GW experiences synesthetic colors in response to different types of stimuli (names of personal acquaintances, emotional words), their connection shaped by personal life experience and affective (valenced) reactions toward these stimuli. As will be argued below, such connections, shaped over a lifetime, might be accompanied by connections that change with a given situation.

Before moving to the following sections, it seems constructive finalizing this section by noting the basic distinction between emotion and affect. Novices to emotion research tend to meddle with various concepts important to emotion or affect theories. Affective scientists, indeed, make very clear distinctions between affect, emotion, and other affective phenomena. Affect basically refers to mental states characterized by evaluative feelings. Affective states represent a broad and overarching collection of “affective subtypes” such as emotions, moods, attitudes, preferences, interpersonal stances, and affect dispositions (Scherer, 2005). Both psychophysiological reactions and cognitive appraisal influence the affective experience to different degrees. By inference, such reactions and appraisals could be relevant to affect-related or affect-modulated synesthesias. Before considering temporary aspects of these possibilities (stable vs. dynamic affect relationships), we account for ideas on the origin of synesthesia because they influence our understanding on how idiosyncratic and stable synesthesia may occur.

## Cross-wiring in synesthesia and crossmodal processing in non-synesthetes

According to aetiological models, synesthetic experiences emerge from co-activation of otherwise independent sensory, closed modular systems (Baron-Cohen and Harrison, 1993). This co-activation might emerge from associative learning (Galton, 1880; Stevenson et al., 1998) disinhibited feedback (Grossenbacher and Lovelace, 2001), enhanced neural connectivity (Rouw and Scholte, 2007; Bargary and Mitchell, 2008), and/or neural cross-talk (Hubbard and Ramachandran, 2001; Ramachandran and Hubbard, 2001b, see also Smilek et al., 2001; Ward, 2013). While none of these models can yet predict in which way these neuronal correlates lead to synesthetic experiences (Deroy and Spence, 2013), it has repeatedly been suggested that such forms of coactivation might also be relevant to crossmodal processing in non-synesthetes (e.g., Simner, 2012 for a recent account, but see Deroy and Spence, 2013). This suggestion is in line with independent notions that synesthesia and crossmodal perception in non-synesthetes might follow a continuum (e.g., Martino and Marks, 2001; Cohen Kadosh and Terhune, 2012; see also Deroy and Spence, 2013 for a critical account on this perspective). Crossmodal mappings common to both synesthetes and non-synesthetes have indeed been reported such as for pitch-lightness (Ward et al., 2006), vision-touch and spatial-numeric interactions (Sagiv and Ward, 2006).

Following this reasoning, and assuming that affective experiences are relevant to the richness of possible crossmodal experiences (Collier, 1996; Palmer et al., 2013b) (and by inference synesthetic experiences as well), we would expect for non-synesthetes (i) that stimuli such as letters, words, numbers, and sounds go systematically with experiences such as colors, brightness, and shapes (see e.g., Collier and Hubbard, 2004) but also (ii) that sensory and affective experiences are linked such that they relate to each other directly, or that sensory experiences are changed by an individual's current affective state.

Studies show systematic associations between color and emotion terms in non-synesthetes from different cultural backgrounds (D'Andrade and Egan, 1974; Johnson et al., 1986; Hupka et al., 1997). The non-synesthetes in Ward (2004) associated color brightness and saturation levels with the pleasantness dimension of emotion, in line with previous findings (Valdez and Mehrabian, 1994). Smiling faces appear brighter than non-smiling faces (Song et al., 2012) and redder faces appear angrier than less red faces (Yasuda et al., 2007). Palmer et al. (2013b) reported systematic mappings between stimuli from different modalities in normal populations. The authors observed significant correlations of both emotional ratings of faces and musical excerpts with emotional ratings of colors chosen to go best with the faces or musical excerpts. These correlations were explained by common affective experiences evoked by or associated with these stimuli. In another study, systematic associations were shown between emotion terms and perceptual aspects such as shape, size and direction, color, music, and sound (Collier, 1996). Collier (p. 30) even concluded that the innate basis for such “affective synesthesias” in non-synesthetes, seemingly present in 2 year olds, “would help explain the universal appeal of the arts.” This conclusion is supported by reports that crossmodal processing involving affective qualities and quantities are important to our aesthetic experience (Palmer et al., 2013a) as well as art production and appreciation (Van Meel, 1994; Rogowska, 2011).

Of additional interest are studies on the body conveying affect in art and other abstract forms of expression. For instance, emotion recognition from stylistic dance (e.g., Dittrich et al., 1996) parallels emotion recognition from non-stylized expressive movement (e.g., Atkinson et al., 2007). Aronoff et al. (1992) observed that emotional attributions to both ballet movements and simple geometric forms are associated with similar shape features (angular and diagonal vs. round) (see Lundqvist et al., 1999 on similar observations for faces). Spatiotemporal cues of arm and whole body movement (size, fluency, joint angle) convey expressed or perceived emotion (Pollick et al., 2001; Atkinson et al., 2007; Visch and Tan, 2009; Dael et al., 2013). Visch and Tan (2009) found that dynamic movement pattern in abstract animated objects influenced emotional responses; slow and direct movement generated sadness, for example, whereas high velocity movements evoked fear and surprise. In the words of Larson et al. (2012; p. 410) “emotion can be effectively and implicitly communicated based on primitive geometric forms that are embedded in many common affective cues.”

The current section showed that crossmodal correspondences are not specific to synesthesia, but that crossmodal processing is common in the general population. For our current purpose, we here focused on crossmodal processing involving affective experiences. If synesthesia and crossmodal processing are based on a partially overlapping aetiology, more synesthesias should be observable linking affect and sensory (including bodily) experiences. As indicated above, such published observations are relatively rare (Cytowic, 1989; Ward, 2004; Callejas et al., 2007; Ramachandran and Brang, 2008; Hochel et al., 2009; Sinke et al., 2012) probably because understudied. For future studies, we would suggest accounting for “emotionality” (discrete emotions, valence) but to also move beyond such more traditional assessments of emotionality by considering a variety of neurophysiological and spatiotemporal body measures as well as cognitive appraisal driving affective experiences.

## The stability and durability of synesthetic experiences questioned

We have seen that studies on affect in synesthesia dealt with discrete emotions (so far one report to the authors' knowledge: Ramachandran and Brang, 2008), affective valence (Ward, 2004), affective preferences (Callejas et al., 2007), and pain (Dudycha and Dudycha, 1935). Intuitively, the likely assumption would be that affective cross-links would be stable, durable and idiosyncratic (but see Ward, 2004), as commonly assumed for crossmodal experiences in developmental synesthesia. Yet, as will be shown, synesthetic experiences might disappear, new ones may develop in later life, and some might depend on the context, with possibly synesthetic experiences involving affect.

Changes to synesthetic experiences have been suggested since the beginning of the last century with a potentially higher prevalence of synesthesia in children than in adults (Alford, 1918; Riggs and Karwoski, 1934). Over a 12 months period, new synesthetic experiences were reported from children between 6 and 8 years old (Simner et al., 2009). As will be shown, new synesthetic experiences do also occur in later life. These experiences refer to new synesthetic couplings when encountering new stimuli. For instance, in the case of object personification synesthesia, stable personalities are experienced for familiar but also for novel objects, even after a single encounter (Smilek et al., 2007). When it comes to lexical material, four individuals out of 192 reported synesthetic experiences when exposed to language they do not understand (Rich et al., 2005). In addition, these same authors reported on a synesthete whose mother tongue was English and who had learned Modern Greek as an adult. This synesthete showed comparable synesthetic experiences according to the shape of the letters in the two different alphabets (as compared to the letter's similarity in pronunciation) (see also Witthoft and Winawer, 2006; Simner et al., 2011 for synesthetic experiences in different languages). A recent study showed that it took only a 10-min writing exercise for grapheme-color associations to be transferred to novel graphemes, here Glagolitic letters, characters from an ancient language (Mroczko et al., 2009).

Although synesthetic experiences are generally stable from early childhood, there is some evidence to suggest that new couplings can be formed with new stimuli over even short periods of time, or be mediated by context (encountering new persons, learning new skills). As noted before, the inducer is not the “physical” stimulus for the synesthetic experience itself, but how an object or experience is interpreted (e.g., Simner, 2007). Most of the time, an object (e.g., letter) is likely interpreted in the same way and by inference leads to the same concurrent. Yet, as outlined above, life situations might be appraised differently across time leading to potential changes to the synesthetic experience. If a certain “dynamism” applies to synesthesias involving affect alike, one might expect changes in the synesthetic experience with relatively lasting changes to affective measures (e.g., changed preferences and likings for persons, objects, sounds, activities). However, as will be considered in the next section, changes in the synesthetic experience involving affect might also be short lasting.

## Temporary changes to crossmodal processing when involving affect

The literature on crossmodal processing would hint at relatively short-lasting changes to synesthetic experiences involving affect. More specifically, we refer (i) to situations differing in their current affect such as the exposure to pleasant or unpleasant sensory stimulations (e.g., odor, images) and (ii) temporary changes to a person's mood (failing exams, break-up of a romantic relationship, death of a kin). While possible synesthetic and crossmodal links involving affect have been outlined above, we are dealing here with the possibility that crossmodal experiences change in quality or quantity as a function of how the individual affectively perceives the sensory experience at the time of testing or as a function of the affective state the individual is at the time of testing.

Studies assessed altered sensory perceptions after the manipulation of participants' affective states typically by comparing performance after positive/negative mood induction as compared to a neutral state induction using various materials. Siegel and Stefanucci (2011), for instance, reported that participants considered sounds to be significantly louder after a negative mood induction as compared to participants after a neutral mood induction. Noise-induced anger biased the perception of red for an ambiguously colored red-blue computer screen (Fetterman et al., 2012). Affective word evaluations primed brightness perceptions of gray squares congruent with the hypothesized metaphor (negative = dark; positive = light) (Meier et al., 2007)—a result that was replicated after listening to emotion-evoking musical excerpts (Lindsen and Bhattacharya, 2012). Chen and Dalton (2005) found that anxious individuals and neurotic individuals detected pleasant and unpleasant odors faster than neutral odors. Moreover, in men, the intensity of the odors was perceived to be stronger when being in an emotional (irrespective of valence) as compared to a neutral state. In another study, olfactory sensitivity was negatively affected after having seen unpleasant pictures, although odors were subjectively rated to be more intense (Pollatos et al., 2007). Thus, affective states seem to modify sensory experiences temporarily.

Additional studies further strengthen the notion that modifications can occur for stimuli of varying complexity (e.g., primary visual analysis, size estimations, processing of social cues) and at early or later processing stages (e.g., Phelps et al., 2006; Bocanegra and Zeelenberg, 2009; Schmitz et al., 2009; Stefanucci et al., 2011; Rossi and Pourtois, 2013). When relatively “simple” target features were concerned, brief presentation of a fearful face as compared to a neutral one resulted in superior orientation sensitivity for images of low spatial frequency and attenuated orientation sensitivity for images of high spatial frequency (Bocanegra and Zeelenberg, 2009). Phelps et al. (2006) observed enhanced contrast sensitivity for Gabor patches (early visual processing) subsequent to fear induction. Also, visual field processing is narrowed when in a negative mood and widened when in a positive mood (Schmitz et al., 2009; see also Gable and Harmon-Jones, 2010). In an ERP study, Rossi and Pourtois (2013) found that effective filtering (perceptual encoding) of peripheral task-irrelevant distractors was abolished when individuals were in a negative mood. Another study showed that height is overestimated when looking at high vs. low arousing pictures and that this effect is enhanced when individuals up-regulated their emotional experience (Stefanucci and Storbeck, 2009). Moreover, when standing on a skateboard on top of a hill, participants who were scared as compared to those who were not judged the hill to be steeper (Stefanucci et al., 2008). Anxious participants who were in the dark judged auditory targets to be closer than non-anxious participants (Gagnon et al., 2013).

When considering “more complex” target features, Anderson et al. (2011) found that participants having experienced an unpleasant affect as compared to a neutral situation had a processing bias for emotional faces in a binocular rivalry paradigm. In lexical decision and word naming experiments, emotional state facilitated responses to words categorically related to the induced emotion (i.e., happy vs. sad words) (Niedenthal and Setterlund, 1997). Ferraro et al. (2003) showed that happy and sad mood inductions (listening to classical music) resulted in faster responding to happy and sad words, respectively. In another series of studies, a decreased holistic processing bias for faces has been reported subsequent to a negative as compared to positive and neutral mood induction (Curby et al., 2011; see also Lynn et al., 2012). Yet, questioning the role of valence, when having undergone an arousal induction procedure, participants yielded a global vs. local processing bias for geographical map information, irrespective of the valence of the arousal situation (Brunyé et al., 2009).

The current section showed that perception changes as a function of individuals' affective state, but also as a function of the affective properties of the material. If these findings apply to synesthesia, we could expect to observe slight changes to the synesthetic experiences in analogy to the observations described here. Changes are potentially subtle and may only become recognizable when of sufficient magnitude. The studies reviewed here would point to quantitative changes rather than qualitative changes: experiences were larger, closer, darker, steeper and faster. For synesthetes, one could thus expect short-lasting quantitative changes such as the synesthetic colors being lighter when in a positive state (Meier et al., 2007; Lindsen and Bhattacharya, 2012), or the months being spatially localized more closely when in an anxious state (Gagnon et al., 2013). These predictions on a mainly quantitative level would also fit with synesthetes' own experience that the synesthetic experience remains the same. If synesthetes would experience major qualitative changes, these would have certainly been reported (but obviously the possibility should not be excluded), while quantitative ones might mainly be too subtle to be overtly recognized as such by the individual (see also Deroy and Spence, 2013).

In this regard, we would like to finish this section with reference to a promising report on a synesthete published in 1935 by Dudycha and Dudycha. Among other synesthetic crossmodal correspondences, their participant reported auditory—shape synesthesias. When hearing the same person speaking in a rasping voice, the accompanying shapes would be larger and sharper. Also, the shapes would be larger with increasing harshness of the same voice. Here the synesthete was aware of these relationships. The authors write the “subject observes the photism which is caused by her own speaking voice, and states that whenever she observes that it is becoming rough or jagged she modulates her voice so as to make the photism smooth and rod-like.” (p. 63).

## Conclusions

In the current article, we aimed to provide a rationale for the probable role of affect in developmental synesthesia. We reviewed the literature on synesthesia (in particular relevant to affective processing) on the one hand and introduced some general theoretical concepts and empirical findings from the emotion and crossmodal literature on the other hand. The motivation for this endeavor relates to the relatively minimal attention affect has received in the published literature on synesthesia. Indeed, the synesthetic experience might not be affective to the synesthete who is used to these experiences (Sinke et al., 2012). Yet, when briefly reviewing the literature on crossmodal processing in synesthesia, modular (e.g., on discrete emotions) and non-modular (e.g., cognitive appraisal) emotion theories, as well as crossmodal processing (including affect) in the general population, two major predictions emerged. Firstly, since cognitive appraisal determines the form and intensity of the affective experience, it should also to some extent determine potential idiosyncrasies of or changes to the synesthetic experience. In these cases, the synesthetic experience involving affect would be relatively long-lasting if the affective appraisal is likely to remain stable (e.g., very unpleasant odors) or might change depending on changes in affective appraisal (e.g., from liking to disliking of particular persons). Secondly, synesthesias involving affect might be subject to temporary changes of relatively short duration according to the affective evaluation of a stimulus (e.g., a less pleasant odor being judged more intense) or the current affective state of the individual (e.g., an anxious person perceives the hill as more steep). Here, changes to the synesthetic experience are proposed to be mainly quantitative rather than qualitative.

We suggest that such future studies might also benefit from the consideration of appraisal accounts of emotion and affect (see for example Fontaine et al., 2007) as well as psychological theories suggesting associative mechanisms by which prior affective (valenced) experiences with objects or concepts can explain current preferences (Palmer and Schloss, 2010).

## Author contributions

Development of basic article idea and structure Nele Dael, Christine Mohr. Literature search, analysis, and writing Nele Dael, Guillaume Sierro, Christine Mohr

### Conflict of interest statement

The authors declare that the research was conducted in the absence of any commercial or financial relationships that could be construed as a potential conflict of interest.
